# A Multi-Center Study to Evaluate the Performance of Phage Amplified Biologically Assay for Detecting TB in Sputum in the Pulmonary TB Patients

**DOI:** 10.1371/journal.pone.0024435

**Published:** 2011-09-08

**Authors:** Changtai Zhu, Zhenling Cui, Ruijuan Zheng, Hua Yang, Ruiliang Jin, Lianhua Qin, Zhonghua Liu, Jie Wang, Zhongyi Hu

**Affiliations:** 1 Shanghai Key Laboratory of Tuberculosis, Shanghai Pulmonary Hospital, School of Medicine, Tongji University, Shanghai, People's Republic of China; 2 Department of Laboratory Medicine, Changzhou Tumor Hospital Soochow University, Changzhou, People's Republic of China; Los Angeles Biomedical Research Institute, United States of America

## Abstract

**Objective:**

To evaluate the performance of phage amplified biologically assay (PhaB) for detecting tuberculosis (TB) in sputum in the pulmonary tuberculosis (PTB) patients.

**Methods:**

Shanghai Tuberculosis Key Laboratory of Shanghai Pulmonary Hospital participated in the project in collaboration with the laboratories of six hospitals and a total of 1660 eligible participants (1351 PTB patients and 309 non-TB patients) were included in the study. The sputum samples from the participants were detected by smear microscopy, PhaB, and Löwenstein-Jensen (L-J) culture method, respectively.

**Results:**

The overall sensitivity of PhaB were higher than that of L-J culture and smear microscopy (*p*<0.05). The sensitivity of PhaB for detecting smear-negative specimens was obviously higher than that of L-J culture (*p*<0.05). Compared with L-J culture, the overall sensitivity, specificity, PPV, NPV, ACC and Kappa value of PhaB were 98.4 (95% Cl: 96.9–99.3), 71.6 (95% Cl: 68.4–74.6), 67.7, 98.7, 81.7% and 0.643, respectively. The detection median time of PhaB only needed 48 hours, which was significantly less than that (31 days) of L-J culture method.

**Conclusion:**

PhaB method is a rapid and sensitive method for detecting TB in sputum in PTB patients; especially for the diagnosis of smear-negative PTB, PhaB method is obviously more sensitive than L-J culture method.

## Introduction

**Figure 1:**
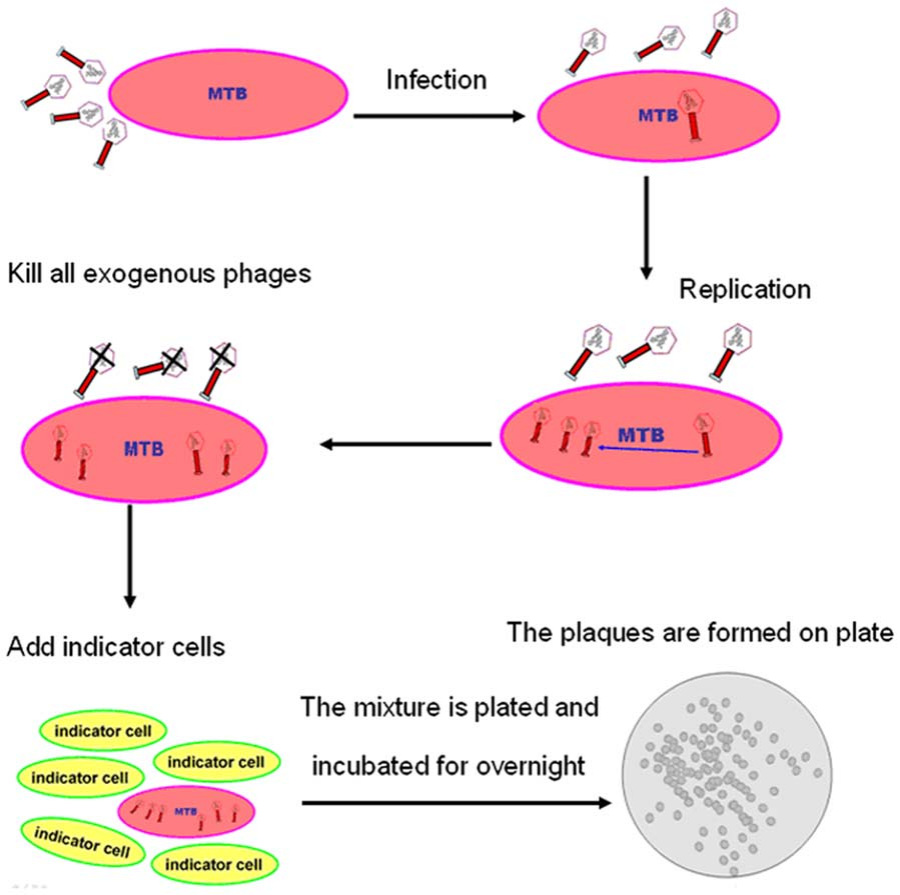
The primary procedures of PhaB method for detection of TB.

Tuberculosis (TB) is a common and deadly infectious disease that is caused by *Mycobacterium tuberculosis* (MTB). The World Health Organization estimated that, the global burden of disease caused by TB in 2009 are as follows: 9.4 million incident cases (range, 8.9 million–9.9 million), 14 million prevalent cases (range, 12 million–16 million), 1.3 million deaths among HIV-negative people (range, 1.2 million–1.5 million) and 0.38 million deaths among HIV-positive people (range, 0.32 million–0.45 million). Most cases were in the South-East Asia, African and Western Pacific regions (35, 30 and 20%, respectively) [1]. China is a country of high incidence of TB, with about 4.5 million TB patients, ranking second in the world [1]. Undoubtedly, a rapid and accurate detection is vital to TB diagnosis, treatment, prevention and control, which has been the focus of the study for TB worldwide [2], [3]. Phage amplified biologically assay (PhaB) was an established diagnostic technique for the detection of TB in recent years [4]–[6]. PhaB delicately utilized the ability of mycobacteriophages to infect mycobacteria to detect TB. In detail, mycobacteriophages internalized TB could be protected from chemical inactivation and replicated, which could lyse mycobacteria and the progeny phages were released. And the released phages can lyse fast-growing *Mycobacterium smegmatis* (indicator cell)added subsequently. As a result, on the agar plate would appear translucent plaques. The testing result could be judged from the observation of the plaques [7]. Figure 1 show the primary procedures of PhaB. Recently, PhaB has also been reported to be used in isoniazid, rifampicin and fluoroquinolone susceptibility testing of TB [8]–[13]. However, it still lacks systematic large-scale clinical studies on PhaB. The purpose of the study is to evaluate the performance of phage amplified biologically assay (PhaB) for detecting TB in sputum in the pulmonary tuberculosis (PTB) patients through multi-center cooperation.

## Materials and Methods

### Ethics Statement

All these patients were treated in accordance with the Helsinki Declaration on the participation of human subjects in medical research. The ethics approvals were obtained for this study from Tongji University Ethics Committee, Anhui Province Pulmonary Hospital Province Ethics Committee, Hebei Province Chest Hospital Ethics Committee, Changchun Infectious Diseases Hospital Ethics Committee, Jiangxi Province Chest Hospital Ethics Committee, Tianjin Haihe Hospital Ethics Committee and Chongqing Municipal Public Health Medical Center Ethics Committee. A written informed consent was obtained from each of participants.

### Study participants

**Figure 2:**
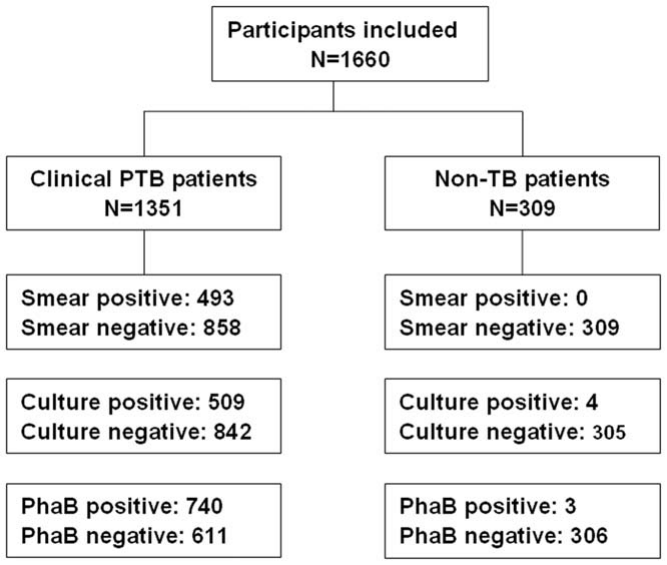
Flow chart of patients included in the study.

Shanghai Pulmonary Hospital in combination with six other hospitals of China (Anhui Province Pulmonary Hospital Province, Hebei Province Chest Hospital, Changchun Infectious Diseases Hospital, Jiangxi Province Chest Hospital, Tianjin Haihe Hospital and Chongqing Municipal Public Health Medical Center) participated in the project. The unified research plans were carefully designed. The clinical diagnoses for PTB were conducted by the physicians according to the guidelines for diagnosis and treatment of pulmonary tuberculosis by the Respiratory Disease Branch of the Chinese Medical Association [14]. The criteria mainly involve symptoms, radiographic findings (chest X-rays or CT scans), tuberculin skin tests, a physical examination, and a medical history. The non-TB patients (having a definite diagnosis of non-TB pulmonary disease) were randomly selected and included in the study for the controls. According to the guidelines recommended by WHO and IUTALD, three sputum samples were collected from each of the participants. The samples were tested immediately for smear microscopy, PhaB and Löwenstein-Jensen (L-J) culture. The physicians were blinded to the results of the mentioned assays and the lab staffs were blinded to the diagnosis of the patients. A total of 1660 eligible participants (1351 consecutive PTB patients and 309 random non-TB patients) were included in the study between Jan 2007 and Dec 2010. The overall characteristics of the enrolled participants were given in Table 1. The flow chart of patients included in the study was shown in Figure 2.

**Table 1:** Characteristics of the clinical PTB patients and non-TB patients.

Characteristics	PTB patients	Non-TB patients
Total Number	1351	309
Age, Median in years (Range, IQR)	34 (10–87, 21–42)	38 (12–85, 22–45)
Male: female	683∶668	167∶142
Known previous history of PTB	97 (7.2%)	0
Received antimycobacterial therapy	42 (3.1%)	0
HIV testing results		
Positive	0	0
Negative	1026	309
Undone	305	0
Classification of disease		
Pneumonia	-	115 (37.22%)
Bronchitis	-	109 (35.28%)
Lung cancer	-	61 (19.74%)
Lung abscess and other	-	24 (7.77%)

IQR: inter quartile range.

### Sputum samples processing

According to the Chinese Anti-tuberculosis Association (CATA) guidelines, the decontamination of sputum specimen was conducted by 4% NaOH-N-acetyl cysteine methods. Based on the degree of sputum viscosity, 2 to 4-fold volume of the liquefier was added. Shook the mixture for 30 s at oscillator and then incubated it at 37°C for 15 min. Added 5 ml liquid medium, centrifuged and washed two times. Subsequently, the supernatant was discarded and the sediment was resuspended in 0.3 ml liquid medium. Finally, 0.1 ml of the mixture suspension was put onto slides for homogenous smear preparation and 0.1 ml of the homogenous suspension was inoculated into L-J slant medium, and 0.1 ml of the remaining was used for PhaB.

### Sputum smear microscopy

The smears were stained by Ziehl-Neelsen (ZN) method according to World Health Organisation (WHO) standard protocol [15].

### Related reagents and materials preparation for PhaB

#### Working phage preparation

The procedures referred to the previous study [13]. *Mycobacterium smegmatis* (ATCC607), kindly offered by China General Microbiological Culture Collection Center (CGMCC), were used for the proliferation of D29 mycobacteriophage (CGMCC) and the filtrate of the proliferation medium was concentrated via filtering using 0.22 µm sterile filter membrane. Subsequently, the phage titer was determined. Finally, the phage was adjusted to 10^9^ PFU/ml for the working concentration (stored at 4°C).

#### The indicator cells preparation

The fast-growing *Mycobacterium smegmatis* was used for indicator cells (acceptance for mycobacteriophage). With the liquid medium, *Mycobacterium smegmatis* being in the exponential growth phase were adjusted to 10^9^ plaque forming unit (PFU)/ml for the working concentration. It was stored at 4°C.

#### Liquid medium

Middlebrook 7H9 culture media were kindly offered by Difico U.S. and nutritional additives (oleic acid, catalase, bovine serum albumin and glucose) were purchased from Sigma. Middlebrook 7H9 liquid medium containing 10% of the nutrition additives was prepared for the experiment.

#### Phage inactivator

With sterile distilled water, ferrous ammonium sulfate (Sigma) was adjusted to 100 mol/L as the Phage inactivator.

### PhaB procedures

The PhaB procedures were referred to the literature [4]. Briefly introduced as follows: 0.2 ml of the suspension of the processed sputum sample, after adding 1 ml liquid medium, was solved and cultured at 37°C for 24 h. 0.1 ml working phage was added and the medium was incubated at 37°C for 1 h. 0.1 ml phage inactivator was added into the medium. Subsequently, the mixture medium was incubated at 37°C for 5 min. Then, added 5 ml liquid medium and 1 ml indicator cells. With 5 ml 1.5% melted agar, the mixture was pour into the plate. After a while, the concretionary plate was incubated at 37°C for overnight. Meanwhile, on each run the positive and negative controls were set for verifying the experimental validation.

### Observation for the results of PhaB

In positive results more than 20 phage plaques in 1–2 mm diameter or the transparent color by merging into many plaques could be seen. In negative results the plaques number appeared in the plate should be less than 20. The negative control should show no plaques and the plaques number of the positive control should be between 20 and 30.

### L-J culture for TB

The procedures of L-J culture and observation for the results were performed according to the CATA guidelines. The description in detail is as follow: 0.2 ml processed specimens were inoculated on the slants of L-J medium (Becton Dickinson) and incubated at 37°C. The slants were inspected every day for first week and then weekly for 8 weeks. All culture positives were identified by ZN staining and standard biochemical identification tests.

### Data analysis

In this study, L-J culture was considered as the gold standard. The performances and comparisons of the test results were performed using Stata version 9 (Statacorp, Texas, USA).

## Results

The sensitivity, specificity, positive predictive value (PPV), negative predictive value (NPV), accuracy (ACC), and Kappa value of PhaB, compared with the reference standard defined by clinical diagnosis, were 54.8 (95% Cl: 52.1–57.5), 99 (95% Cl: 97.2–99.8), 99.6, 33.4, 63% and 0.306, respectively (Table 2). The sensitivity and ACC of PhaB were higher than that of L-J culture and smear method (*p*<0.05).

**Table 2:** Performances of the various methods for testing the clinical sputum specimens compared with the reference standard defined by clinical diagnosis.

	Clinical PTB patients(n = 1351)		Non-TB patients(n = 309)		Testing performances (%, excluded K value)					
	Positive	Negative	Positive	Negative	SN (95% Cl)	SP (95% Cl)	PPV	NPV	ACC	K value
Smear	493	858	0	309	36.5 (33.9–39.1)	100 (98.8–100.0)	100	36	48.3	0.176
L-J culture	509	842	4	305	37.7 (35.1–40.3)	98.7 (96.7–99.6)	99.2	26.6	49	0.178
PhaB	740	611	3	306	54.8 (52.1–57.5)	99 (97.2–99.8)	99.6	33.4	63	0.306

SN: Sensitivity. SP: Specificity. PPV: Positive predictive value. NPV: Negative predictive value.

ACC: Accuracy. Cl: Confidence interval. K value: Kappa value.

The testing results stratified by smear microscopy showed that the sensitivity of PhaB for detecting smear-negative specimens was higher than that of L-J culture (Table 3).

**Table 3:** Comparison of the results of the sputum specimens from clinical PTB patients between L-J culture and the PhaB assay stratified by smear microscopy.

	L-J culture positive	L-J culture negative	PhaB positive	PhaB negative	χ^2^	*p* value
Smear positive(N = 493)	404	89	420	73	1.891	0.167
Smear negative(N = 858)	105	753	320	538	144.57	0.000
Total	509	842	740	611	79.448	0.000

Compared with gold standard of TB diagnosis, namely L-J culture, the overall PhaB sensitivity, specificity, PPV, NPV, ACC and Kappa value were 98.4 (95% Cl: 96.9–99.3), 71.6 (95% Cl: 68.4–74.6), 67.7, 98.7, 81.7% and 0.643, respectively (Table 4); while the counterparts of smear microscopy was 79.4 (95% Cl: 75.6–82.8), 89.4 (95% Cl: 87.2–91.4), 48.0, 87.8, 85.6% and 0.692, respectively (Table 5). The results showed that PhaB for detection of PTB was more sensitive than smear microscopy (*p*<0.05).

**Table 4:** Performance of PhaB for the detection of the sputum specimens from clinical PTB patients compared with L-J culture stratified by smear microscopy.

	The testing results				PhaB performance (%, excluded K value)					
	L-J culturepositive(N = 509)		L-J culturenegative(N = 842)		SN (95% Cl)	SP (95% Cl)	PPV	NPV	ACC	Kvalue
	PhaBpositive	PhaBnegative	PhaBpositive	PhaBnegative						
Smearpositive(N = 493)	402	2	18	71	99.5 (98.2–99.9)	79.8 (69.9–87.6)	95.7	97.3	95.9	0.853
Smearnegative(N = 858)	99	6	221	532	94.3 (88.0–97.9)	70.7 (67.3–73.9)	30.9	98.9	73.5	0.345
Total(N = 1351)	501	8	239	603	98.4 (96.9–99.3)	71.6 (68.4–74.6)	67.7	98.7	81.7	0.643

**Table 5:** Smear microscopy performance for the detection of the sputum specimens from clinical PTB patients compared with L-J culture method.

	The testing results		Smear microscopy performance (%, excluded K value)					
	L-J culture positive (N = 509)	L-J culturenegative(N = 842)	SN (95% Cl)	SP (95% Cl)	PPV	NPV	ACC	Kvalue
Smear positive(N = 493)	404	89	79.4 (75.6–82.8)	89.4 (87.2–91.4)	48.0	87.8	85.6	0.692
Smear negative(N = 858)	105	753						

As far as the consuming time was concerned, median time of PhaB method is 48 hours (inter quartile range [IQR]: 42–51 hours), whereas that of L-J culture method would take 31.0 days (IQR: 28–35 days). The detection time of PhaB method was significantly less than that of L-J culture method (*p*<0.05).

## Discussion

Previous small samples reports showed that the PhaB could be used as a tool of PTB diagnosis indicating that PhaB might be of potential clinical value [16]–[19]. In this study, based on the previous studies, we set up the standard PhaB procedures. Furthermore, in order to fulfill to the clinical application in large scale, we made a set of kits (consisting of working phage 1 bottle, indicator cell 1 bottle, liquid medium 1 bottle, phage inactivator 1 bottle and the controls 2 bottles), which was very convenient for routine use in common clinical laboratories. To evaluate the performance of the kits, we combined the six laboratories in China, as the unified designed project, 1660 sputum samples were detected by PhaB and culture method, respectively.

In this study, the sensitivity of PhaB, L-J culture and smear method in comparison with the reference standard defined by clinical diagnosis were 54.8, 37.7, and 36.5%, respectively, suggesting that PhaB had higher sensitivity against the other two methods. Whereas, according to the testing results stratified by smear microscopy, out of 858 smear-negative specimens, 320 (37.3%) were positive by PhaB and 105 (12.2%) were confirmed to be positive by L-J culture. Apparently, the sensitivity of PhaB was higher than that of L-J culture. Compared with gold standard of TB diagnosis, namely L-J culture, the overall sensitivity of PhaB was 98.4%, while that of smear microscopy was 79.4%, indicating that the overall sensitivity of PhaB was higher than smear microscopy. Based on the above facts, it could be concluded that the sensitivity of PhaB was higher than that of L-J culture and smear microscopy, especially for detection of smear-negative specimens.

It is the fact that, due to too slow growth, some *M. TB* clinical strains may lead to false negative results by using L-J culture method [20]; however, that doesn't affect the detection validation of PhaB method. It is no wonder that 45.2% (611/1351) patients were negative by using PhaB method. On one hand, phage could only infect live mycobacteria, if they were killed via the antimicrobials treatment, the results would reveal negative [19]. On the other hand, the intermittent discharged mycobacteria could affect the test results. So, multiple detections in different dates and collecting qualified specimens are also very important to PhaB method.

As for the evaluation of the specificity of PhaB, the discrepant results appeared between using clinical diagnosis as standard and referring to L-J culture. Compared with L-J culture, the overall specificity of PhaB was only 71.6%; while using the clinical diagnosis as a reference standard, the specificity was 99%. For several decades, L-J culture has been considered as a gold standard for the evaluation of new assays of TB diagnosis. Undoubtedly, a perfect gold standard testing method should be 100% specificity and 100% sensitivity. Unfortunately, L-J culture isn't an ideal gold standard testing method for TB diagnosis, mainly due to its low sensitivity. Therefore, we predicted that in this study some true positive results that could be wrongly tested by PhaB might be mistaken for true negative results by L-J culture. Surely, such a mistake would lead to the descent of specificity of PhaB in comparison with the true specificity value. Of course, considering clinical comprehensive diagnosis as a reference standard has some limitations, due to its unknown accuracy. However, a more effective gold reference standard is expected to be developed in the future, which is vital to the evaluation of new diagnostic tests for TB.

Theoretically, mycobacteriophages are only able to infect Mycobacteria, which could ensure the high specificity of PhaB method. However, PhaB method might produce some very little false positive or false negative results. The cross reaction (no-specificity of phage) could occur between MTB and other Mycobacteria including *Mycobacterium smegmatis*, *Mycobacterium bovis*, *Mycobacterium Kansas*, and *Mycobacterium butyrate* [21], [22]. This is the main reason causing the PhaB false-positive in clinical specimens. In spite of this, the proportion of the other Mycobacteria was very small, so the overall experiments' specificity was still higher. In addition, some technical factors could influence PhaB method, such as: the sputum pre-treatment quality (incomplete liquefaction can stop the phage infection), the infection time, concentration and conditions of mycobacteriophage, and inactivator concentration and conditions. We recommended that, 4% NaOH-N-acetyl cysteine methods should be taken as the sputum decontamination, 10^9^ PFU/ml D29 mycobacteriophage at 37°C for 60 min as mycobacteriophage infection conditions, 100 mmol/ml at 37°C for 5 min as inactivator concentration and conditions. In addition, we also noticed that a majority of the results of PhaB positive while L-J culture negative had smaller plaques number (median: 33 PFU, IQR: 22–57 PFU) in this study. However, amongst the cases of L-J culture positive while PhaB negative, we couldn't discern any valuable clues.

As far as consuming time was concerned, median time of PhaB method needed 48 hours (IQR: 42–51 hours), whereas that of L-J culture method would consume 31.0 days (IQR: 28–35 days). The detection time of PhaB method was significantly less than that of L-J culture method.

In brief, PhaB method is a rapid and sensitive method for detecting TB in sputum in pulmonary TB patients; particular in the diagnosis of smear-negative PTB, PhaB method is obviously more sensitive than L-J culture method.
